# Tris(2-carbamoylguanidinium) hydrogen fluoro­phospho­nate fluoro­phospho­nate monohydrate

**DOI:** 10.1107/S1600536811051683

**Published:** 2011-12-07

**Authors:** Jan Fábry, Michaela Fridrichová, Michal Dušek, Karla Fejfarová, Radmila Krupková

**Affiliations:** a Institute of Physics of the Czech Academy of Sciences, v. v. i., Na Slovance 2, 182 21 Praha 8, Czech Republic; b Department of Inorganic Chemistry, Faculty of Science, Charles University in Prague, Hlavova 2030, 12843 Prague 2, Czech Republic

## Abstract

The title structure, 3C_2_H_7_N_4_O^+^·HFPO_3_^−^·FPO_3_^2−^·H_2_O, contains three independent 2-carbamoylguanidinium cations, one fluoro­phospho­nate, one hydrogen fluoro­phospho­nate and one water mol­ecule. There are three different layers in the structure that are nearly perpendicular to the *c* axis. Each layer contains a cation and the layers differ by the respective presence of the water mol­ecule, the hydrogen fluoro­phospho­nate and fluoro­phospho­nate anions. N—H⋯O hydrogen bonds between the guanylurea mol­ecules that inter­connect the mol­ecules within each layer are strong. The layers are inter­connected by strong and weak O—H⋯O hydrogen bonds between the anions and water mol­ecules, respectively. Inter­estingly, the configuration of the layers is quite similar to that observed in 2-carbamoylguanidinium hydrogen fluoro­phospho­nate [Fábry *et al.* (2012). *Acta Cryst.* C**68**, o76–o83]. There is also present a N—H⋯F hydrogen bond in the structure which occurs quite rarely.

## Related literature

For the related structures 2-carbamoylguanidinium hydrogen fluoro­phospho­nate and bis­[guanylurea)(1+)] fluoro­phospho­nate dihydrate, see: Fábry *et al.* (2012*a* ▶,*b* ▶). For the related compound 2-carbamoylguanidinium hydrogen phosphite and its physical properties, see: Fridrichová *et al.* (2010*a* ▶,*b* ▶); Kroupa & Fridrichová (2011 ▶). For the applied values of the constraints for water mol­ecules, see: Allen (2002 ▶). For preparation of the precursors, see Ostrogovich (1911 ▶); Schülke & Kayser (1991 ▶); Scoponi (1991 ▶). For the involvement of fluorine in hydrogen bonds, see: Dunitz & Taylor (1997 ▶); Krupková *et al.* (2002 ▶). For the denomination of the hydrogen bonds, see: Desiraju & Steiner (1999 ▶). For the extinction correction, see: Becker & Coppens (1974 ▶).
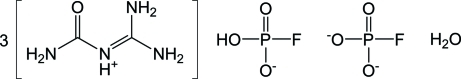


## Experimental

### 

#### Crystal data

3C_2_H_7_N_4_O^+^·HFO_3_P^−^·FO_3_P^2−^·H_2_O
*M* *_r_* = 524.3
Triclinic, 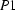

*a* = 6.7523 (3) Å
*b* = 8.2926 (3) Å
*c* = 9.7297 (4) Å
α = 100.630 (3)°
β = 90.885 (3)°
γ = 99.168 (3)°
*V* = 528.05 (4) Å^3^
*Z* = 1
Mo *K*α radiation
μ = 0.30 mm^−1^
*T* = 297 K
0.60 × 0.45 × 0.40 mm

#### Data collection

Oxford Diffraction Xcalibur Gemini ultra diffractometer
Absorption correction: multi-scan (*CrysAlis PRO*, Oxford Diffraction, 2010 ▶) *T*_min_ = 0.852, *T*_max_ = 0.888
8499 measured reflections
4769 independent reflections
4203 reflections with *I* > 3σ(*I*)
*R*_int_ = 0.018

#### Refinement

*R*[*F* ^2^ > 2σ(*F* ^2^)] = 0.030
*wR*(*F* ^2^) = 0.068
*S* = 1.52
4769 reflections
352 parameters
22 restraints
H atoms treated by a mixture of independent and constrained refinement
Δρ_max_ = 0.19 e Å^−3^
Δρ_min_ = −0.16 e Å^−3^
Absolute structure: Flack (1983) ▶, 2216 Friedel pairs
Flack parameter: 0.08 (6)

### 

Data collection: *CrysAlis PRO* (Oxford Diffraction, 2010 ▶); cell refinement: *CrysAlis PRO*; data reduction: *CrysAlis PRO*; program(s) used to solve structure: *SIR97* (Altomare *et al.*, 1999) ▶; program(s) used to refine structure: *JANA2006* (Petříček *et al.*, 2006 ▶); molecular graphics: *PLATON* (Spek, 2009 ▶), *DIAMOND* (Brandenburg, 2010 ▶) and *Origin* (OriginLab, 2000 ▶); software used to prepare material for publication: *JANA2006*.

## Supplementary Material

Crystal structure: contains datablock(s) global, I. DOI: 10.1107/S1600536811051683/zj2040sup1.cif

Supplementary material file. DOI: 10.1107/S1600536811051683/zj2040Isup2.smi

Structure factors: contains datablock(s) I. DOI: 10.1107/S1600536811051683/zj2040Isup3.hkl

Additional supplementary materials:  crystallographic information; 3D view; checkCIF report

## Figures and Tables

**Table 1:** The hydrogen-bond (Å, °) pattern in the title structure Atoms N1—N4, N5—N8 and N9—N12 are situated in the first, second and third layers, respectively. The atoms in the blocks comprise the corresponding atoms in the respective layers: *e.g.* N2—H2*n*2⋯O1, N5—H2*n*5⋯O2, N10—H1*n*10⋯O3.

*D*—H⋯*A*	*D*—H	H⋯*A*	*D*⋯*A*	*D*—H⋯*A*
O11—H1*o*11⋯O21	0.80 (3)	1.72 (4)	2.515 (3)	174 (4)
O*w*—H1*ow*⋯O13	0.82 (2)	1.957 (19)	2.754 (3)	164 (2)
O*w*—H2*ow*⋯O22^i^	0.82 (2)	2.253 (17)	3.037 (3)	160 (3)
O*w*—H2*ow*⋯O23^i^	0.82 (2)	2.41 (3)	3.023 (3)	132 (3)
				
N1—H1*n*1⋯O*w*	0.860 (18)	2.302 (15)	3.022 (4)	141 (2)
N1—H2*n*1⋯O1^ii^	0.861 (17)	2.24 (2)	2.795 (3)	122.4 (15)
N1—H2*n*1⋯O23^iii^	0.861 (15)	2.431 (14)	3.141 (3)	140.3 (18)
N6—H2*n*6⋯O2^ii^	0.860 (17)	2.23 (2)	2.750 (3)	119.0 (15)
N6—H2*n*6⋯O12^iv^	0.860 (17)	2.491 (15)	3.200 (3)	140.3 (17)
N6—H1*n*6⋯O11	0.861 (19)	2.28 (2)	3.141 (3)	174 (2)
N9—H2*n*9⋯O21^iv^	0.860 (17)	2.08 (2)	2.916 (2)	164 (2)
N9—H1*n*9⋯F2^v^	0.860 (7)	2.479 (19)	3.096 (2)	129 (2)
N9—H1*n*9⋯O3	0.860 (7)	2.01 (2)	2.634 (2)	129 (2)
				
N2—H2*n*2⋯O*w*	0.861 (16)	2.12 (2)	2.894 (3)	150 (2)
N2—H1*n*2⋯O1	0.861 (12)	1.94 (2)	2.618 (3)	135 (2)
N5—H2*n*5⋯O13	0.862 (19)	2.07 (2)	2.906 (3)	165 (2)
N5—H1*n*5⋯O2	0.860 (7)	2.03 (2)	2.646 (3)	128 (2)
N10—H1*n*10⋯O21^iv^	0.860 (13)	2.595 (10)	3.318 (3)	142.5 (17)
N10—H2*n*10⋯O3^vi^	0.861 (13)	2.186 (14)	2.750 (3)	122.9 (11)
N10—H2*n*10⋯O22	0.861 (13)	2.524 (13)	3.209 (3)	137.1 (11)
				
N3—H1*n*3⋯O23^iii^	0.89	1.95	2.770 (3)	153
N7—H1*n*7⋯O12^iv^	0.89	1.93	2.807 (3)	166
N11—H1*n*11⋯O22	0.89	1.93	2.804 (3)	166
				
N4—H1*n*4⋯O23^vii^	0.860 (14)	2.386 (16)	3.170 (3)	152 (2)
N4—H2*n*4⋯O*w*^iv^	0.86 (2)	2.32 (2)	3.173 (3)	176 (2)
N8—H2*n*8⋯O13^iv^	0.857 (16)	2.024 (16)	2.877 (3)	173.8 (15)
N8—H1*n*8⋯O12^viii^	0.860 (10)	2.179 (10)	3.034 (3)	173 (3)
N12—H1*n*12⋯O21	0.859 (11)	2.115 (16)	2.972 (2)	175 (3)
N12—H2*n*12⋯O22^ii^	0.859 (11)	2.198 (11)	3.031 (3)	163 (3)

Symmetry codes: (i) 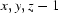; (ii) 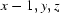; (iii) 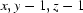; (iv) 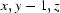; (v) 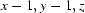; (vi) 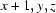; (vii) 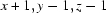; (viii) 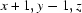.

**Fig. 1.**
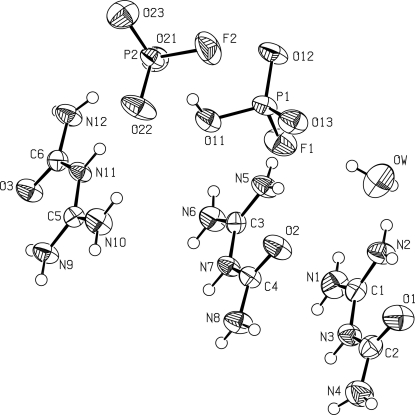
View of the constituent molecules and ions of the title structure; the displacement ellipsoids are depicted at the 50% probability level (Spek, 2009).

**Fig. 2.**
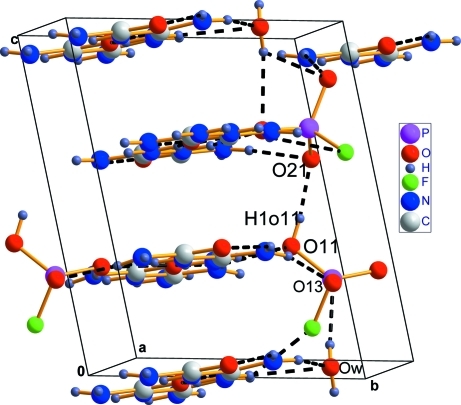
View of the unit cell of the title structure (Brandenburg, 2010.)

**Fig. 3.**
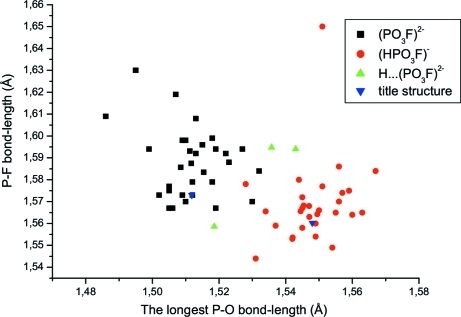
Plot of P—F vs. the longest P—O bond lengths in the molecules of hydrogen fluorophosphonate and fluorophosphonate. The plot was constructed by Origin (OriginLab, 2000). The title anions are symbolized by blue triangles; the left triangle corresponds to the fluorophosphonate while the right one to the hydrogen fluorophosphonate in the structure. For the list of the structures that entered into this plot, see the deposited material or Fábry et al. (2012a).

**Fig. 4.**
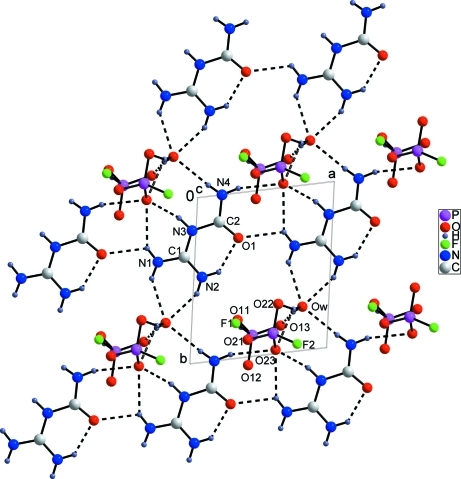
View of the hydrogen bond pattern within the layer with z \sim 0.0 of the title structure. This layer contains the water molecules (Brandenburg, 2010).

**Fig. 5.**
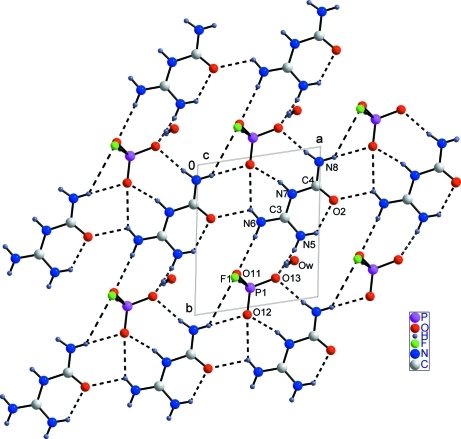
View of the hydrogen bond pattern within the layer with z ~0.3 of the title structure. This layer contains hydrogen fluorophosphonate (Brandenburg, 2010). Compare with Fig. 7.

**Fig. 6.**
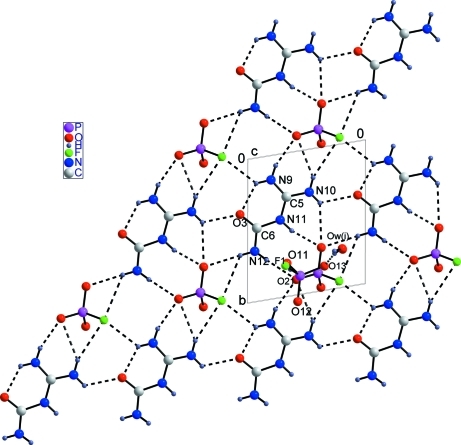
View of the hydrogen bond pattern within the layer with z ~0.6 of the title structure. Symmetry code: (i) x, y, z + 1. This layer contains fluorophosphonate (Brandenburg, 2010).

**Fig. 7.**
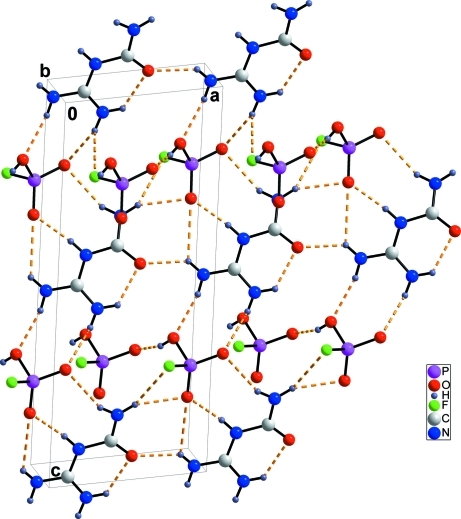
Section of guanylurea hydrogen fluorophosphonate (Fábry et al., 2012a) showing the hydrogen-bond pattern (Brandenburg, 2010). Compare with Fig. 5.

**Fig. 8.**
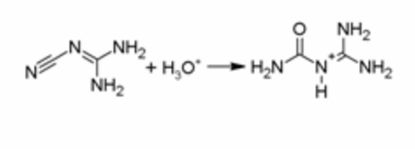
Scheme of the preparation of guanylurea.

## supplementary crystallographic information

### Comment

Preparation of the title structure was stimulated by the study of 2-carbamoylguanidinium hydrogen fluorophosphonate (Fábry *et al.*, 2011*a*) and the isostructural 2-carbamoylguanidinium hydrogen phosphite (Fridrichová *et al.*, 2010*a*). It turned out that both compounds form mixed crystals (Fábry *et al.*, 2011*a*). 2-carbamoylguanidinium hydrogen phosphite (Fridrichová *et al.*, 2010*a*) shows interesting optical properties (Fridrichová *et al.*, 2010*b*; Kroupa & Fridrichová, 2011). These compounds were grown from equimolar solutions of the cations and anions.

In order to extend the study of the system guanylurea - fluorophosphonate there have been carried out experiments on the crystal growth from the solutions of different molar ratios of the 2-carbamoylguanidinium cation and the fluorophosphonate. One of such experiments resulted in preparation of the title structure and the other one in preparation of two polymorphs of bis[guanylurea)(1+)] fluorophosphonate dihydrate (Fábry *et al.*, 2011*b*).

The title structure contains three independent 2-carbamoylguanidinium molecules, one fluorotrixophosphate, one hydrogen fluorophosphonate and the water molecule (Fig. 1). The layers, which are nearly perpendicular to the *c* axis, are interconnected by the O—H···O hydrogen bonds between the fluorophosphonate, the hydrogen fluorophosphonate and the water molecules (Fig. 2; Tab. 1). The hydrogen bond between the fluorophosphonate and the hydrogen fluorophosphonate is quite strong (Desiraju & Steiner, 1999) in contrast to the weaker ones between the water molecule and the O atoms of the hydrogen fluorophosphonate and the fluorophosphonate.

The geometrical parameters of both anions are in accordance with the observed values in these anions in other structures (Fábry *et al.*, 2011*a*; deposited material at the end of the CIF). Fig. 3 shows that the [PO_3_F]^2-^ anion in the title structure is situated in the region where the anion can be affected as an acceptor of the hydrogen bond, *i. e.* close to the intermediate region of [H···PO_3_F]^-^. Indeed, the involvement of O21 in the strong hydrogen bond O11-H1o11···O21 is accompanied by the concomitant shortening of the P—F bond length, *i.e.* of P2—F2 in the present case. A similar, even more pronounced effect has recently been observed in bis[guanylurea)(1+)] fluorophosphonate dihydrate (Fábry *et al.*, 2011*b*).

On the other hand, the position of [HPO_3_F]^-^ corresponds to the full hydrogenation of the anion. This is in accordance with the refined position of the hydroxyl hydrogen that is situated close to the donor oxygen despite of the short O—H···O hydrogen bond between both anions.

The 2-carbamoylguanidinium cations within each layer are interconnected between themselves by the N—H···O hydrogen bonds (Fig. 2; Figs. 4–6). These hydrogen bonds are bent with 135 (2)° as maximum for N2-H1*n*2···O1 (Tab. 2). Moreover, the 2-carbamoylguanidinium cations which are situated in respective layers are also interconnected by the N—H···O hydrogen bonds with water molecules (layer 1; Fig. 4), hydrogen fluorophosphonates (layer 2, Fig. 5) and fluorophosphonates (layer 3, Fig. 6). It is interesting that the secondary amines form stronger N—H···O hydrogen bonds than the primary ones (Tab. 2). Similarly as in 2-carbamoylguanidinium hydrogen fluorophosphonate (Fábry *et al.*, 2011*a*) all the amine H atoms are involved in the hydrogen bonds (Tab. 2) with H···O(acceptor) spanning the range 1.93 – ~2.40 Å. This means that these hydrogen bonds are considered as the strong and weak hydrogen bonds (Desiraju & Steiner, 1999).

The title structure is rather unusual for presence of a rare N—H···F hydrogen bond (Dunitz & Taylor, 1997; Krupková *et al.*, 2002) where F belongs to the hydrogen fluorophosphonate (Fig. 5). Nevertheless, all the patterns in the layers are quite similar (Figs. 4–6, Tab. 2). The hydrogen bond motif of the layer 2 (Fig. 5, Tab. 2) corresponds quite well to that of 2-carbamoylguanidinium hydrogen fluorophosphonate (Fábry *et al.* (2011*a*); Fig. 7).

χ^2^ indices regarding the planes fitted through all the non-hydrogen cation's atoms equal to 1048.019, 158.651 and 22.287 for N1, C1, N2, N3, C2, O1, N4; N5, C3, N6, N7, C4, O2, N8 and N9, C5, N10, N11, C6, O3, N12., respectively. Interestingly, in the motif observed in 2-carbamoylguanidinium hydrogen fluorophosphonate (Fábry *et al.*, 2011*a*; Fig. 7), the χ^2^ index equals to 1139.577.

### Experimental

The structures were prepared by neutralization of stoichiometric amounts of guanylurea hydroxide and H_2_PO_3_F. Guanylurea hydroxide was prepared from guanylurea hydrochloride hemihydrate by an exchange reaction on anex.

Guanylurea chloride hemihydrate has been described at the beginning of the 20^-th^ century (Ostrogovich, 1911) and thoroughly characterized by Scoponi *et al.* (1991). It was prepared by acid hydrolysis of cyanoguanidine according to Fig. 8. Diluted water solution (100 ml of water to every 0.1 mol of cyanoguanidine) of equimolar ratios of cyanoguanidine (99%, Sigma-Aldrich) and hydrochloric acid (p.a., Lachema) was gradually heated. After about 45 minutes, when the reaction mixture started boiling, the originally colourless mixture suddenly became greyish and cloudy for a while and then an exothermal process occurred. This process was accompanied by very intense boiling of the reaction mixture. The heating was immediately interrupted and the reaction mixture was placed on a cold magnetic stirrer and it was stirred for another 15 minutes. The liquid which in the meanwhile had turned coulourless again was heated to the boiling point and kept heated for 2 h. Then the excessive water was evaporated under vacuum and a white crystalline product was filtered off. It was purified by recrystallization from water and characterized by powder XRD and found to be identical to the structure JODZOR (Cambridge Crystallographic Database (Allen, 2002; Scoponi *et al.*, 1991). IR spectrum was recorded, too, in order to exclude possibility of contamination of the product by cyanoguanidine. The IR spectrum was in accordance with the compound described by Scoponi *et al.* (1991), whereas the intense doublet of CN^-^ group typical for cyanoguanidine was absent.

The solution of H_2_PO_3_F was prepared from solution of (NH_4_)_2_PO_3_F.H_2_O that passed through the column of catex. (NH_4_)_2_PO_3_F.H_2_O was prepared by the method described by Schülke & Kayser (1991) and the raw material of (NH_4_)_2_PO_3_F.H_2_O prepared by this method was recrystallized in order to get rid of contamination of (NH_4)_H_2_PO_4_. The volume of the eluted solution of H_2_PO_3_F was about 50 ml in both cases. The solutions were put into the evacuated desiccator over P_4_O_10_. The crystals appeared in one week. The crystals deteriorated quickly on air, possibly because of the mother liquor that remained on the surface of the crystals that could react with air humidity. The crystals were put into the special glass capillaries. For the title structure 0.74 g (NH_4_)_2_PO_3_F.H_2_O and 1 g of guanylurea hydroxide was applied. It should be added that the experiments in repeated preparation failed and different crystals have been prepared (Fábry *et al.*, 2012*b*).

### Refinement

All the H atoms were discernible in the difference electron density map. The hydroxyl hydrogen H1o11 of the hydrogen fluorophosphonate was refined freely. The coordinates of the atom P1 have not been refined because of the fixing of the origin in the space group of the title structure. There have been applied the following restraints: Water H atoms were restrained to be distant 0.820 (1) Å from the water oxygen Ow while the H1ow—Ow—H2ow angle was restrained to equal to 107.90 (1)°. [The latter value was retrieved from the neutron diffraction structure determinations contained in the Cambridge Crystal Structure Database (Allen, 2002).] The primary amine hydrogen distances were restrained to 0.860 (1) Å and the angle H1n10-N10-H2n10 was restrained to 120.00 (1) °. The geometry of the secondary amine groups N3, N7 and N11 were constrained as planar (*i. e.* their neighbours were situated in the plane together with the secondary amine groups) with the N—H distances equal to 0.89 Å. The isotropic amine H atoms' displacement parameters have been constrained to 1.2 multiple of *U*_eq_ of the respective carrier N atoms while the *U*_iso_ of the water H atoms equalled to 1.5 multiple of *U*_eq_ of the water oxygen. 2216 Friedel pairs have been used in the refinement. The *x*, *y* and *z* fractional coordinates of P1 atom have been fixed during the refinement.

### Crystal data

**Table:**

3C_2_H_7_N_4_O^+^·HFO_3_P^−^·FO_3_P^2^^−^·H_2_O	*Z* = 1
*M**_r_* = 524.3	*F*(000) = 272
Triclinic, *P*1	*D*_x_ = 1.648 Mg m^−^^3^
Hall symbol: P 1	Mo *K*α radiation, λ = 0.7107 Å
*a* = 6.7523 (3) Å	Cell parameters from 5951 reflections
*b* = 8.2926 (3) Å	θ = 3.0–29.2°
*c* = 9.7297 (4) Å	µ = 0.30 mm^−^^1^
α = 100.630 (3)°	*T* = 297 K
β = 90.885 (3)°	Prism, colourless
γ = 99.168 (3)°	0.60 × 0.45 × 0.40 mm
*V* = 528.05 (4) Å^3^	

### Data collection

**Table:**

Oxford Diffraction Xcalibur Gemini ultra diffractometer	4769 independent reflections
Radiation source: Enhance (Mo) X-ray Source	4203 reflections with *I* > 3σ(*I*)
graphite	*R*_int_ = 0.018
Detector resolution: 10.3784 pixels mm^-1^	θ_max_ = 29.3°, θ_min_ = 3.0°
ω scans	*h* = −9→8
Absorption correction: multi-scan (*CrysAlis PRO*, Oxford Diffraction, 2010)	*k* = −11→11
*T*_min_ = 0.852, *T*_max_ = 0.888	*l* = −13→12
8499 measured reflections	

### Refinement

**Table:**

Refinement on *F*^2^	Hydrogen site location: difference Fourier map
*R*[*F*^2^ > 2σ(*F*^2^)] = 0.030	H atoms treated by a mixture of independent and constrained refinement
*wR*(*F*^2^) = 0.068	Weighting scheme based on measured s.u.'s *w* = 1/[σ^2^(*I*) + 0.0004*I*^2^]
*S* = 1.52	(Δ/σ)_max_ = 0.042
4769 reflections	Δρ_max_ = 0.19 e Å^−^^3^
352 parameters	Δρ_min_ = −0.16 e Å^−^^3^
22 restraints	Extinction correction: B-C type 1 Lorentzian isotropic (Becker & Coppens, 1974)
35 constraints	Extinction coefficient: 700 (200)
Primary atom site location: structure-invariant direct methods	Absolute structure: Flack (1983), 2216 Friedel pairs
Secondary atom site location: difference Fourier map	Flack parameter: 0.08 (6)

### Fractional atomic coordinates and isotropic or equivalent isotropic displacement parameters (Å^2^)

**Table:**

	*x*	*y*	*z*	*U*_iso_*/*U*_eq_	
P1	0.44795	0.87104	0.26806	0.03489 (18)	
O11	0.3358 (3)	0.7684 (2)	0.3693 (2)	0.0531 (7)	
H1o11	0.363 (5)	0.814 (4)	0.449 (4)	0.089 (12)*	
O12	0.4277 (3)	1.0485 (2)	0.2976 (2)	0.0490 (6)	
O13	0.6537 (2)	0.8321 (2)	0.24652 (19)	0.0508 (6)	
F1	0.3223 (2)	0.78577 (19)	0.13057 (16)	0.0606 (5)	
P2	0.59898 (9)	0.87217 (7)	0.70466 (7)	0.03486 (18)	
O21	0.4136 (2)	0.8915 (2)	0.62423 (17)	0.0446 (5)	
O22	0.6261 (3)	0.6971 (2)	0.6955 (2)	0.0591 (7)	
O23	0.6227 (3)	0.9736 (2)	0.84962 (19)	0.0628 (7)	
F2	0.7787 (2)	0.9524 (2)	0.62518 (19)	0.0705 (6)	
N1	0.7279 (3)	0.3585 (3)	−0.0373 (2)	0.0502 (8)	
H1n1	0.687 (4)	0.4524 (16)	−0.032 (3)	0.0603*	
H2n1	0.641 (3)	0.2684 (17)	−0.050 (3)	0.0603*	
N2	1.0574 (3)	0.4809 (3)	−0.0071 (2)	0.0462 (7)	
H1n2	1.1788 (14)	0.464 (3)	0.003 (3)	0.0554*	
H2n2	1.026 (4)	0.5785 (14)	−0.001 (3)	0.0554*	
C1	0.9198 (3)	0.3471 (3)	−0.0309 (2)	0.0359 (7)	
N3	0.9686 (3)	0.1927 (2)	−0.04679 (19)	0.0383 (6)	
H1n3	0.868716	0.106559	−0.058266	0.046*	
C2	1.1648 (3)	0.1594 (3)	−0.0464 (2)	0.0409 (8)	
O1	1.3110 (2)	0.2702 (2)	−0.0345 (2)	0.0550 (6)	
N4	1.1759 (4)	−0.0024 (3)	−0.0644 (3)	0.0555 (9)	
H1n4	1.2923 (18)	−0.032 (3)	−0.066 (3)	0.0666*	
H2n4	1.072 (2)	−0.070 (3)	−0.049 (3)	0.0666*	
N5	0.8507 (3)	0.5738 (3)	0.3262 (2)	0.0412 (7)	
H1n5	0.9772 (8)	0.572 (3)	0.335 (2)	0.0495*	
H2n5	0.804 (4)	0.6619 (18)	0.316 (2)	0.0495*	
N6	0.5269 (3)	0.4422 (3)	0.3139 (2)	0.0442 (8)	
H1n6	0.482 (4)	0.5351 (16)	0.326 (3)	0.0531*	
H2n6	0.441 (3)	0.3523 (17)	0.307 (3)	0.0531*	
C3	0.7199 (3)	0.4378 (3)	0.3164 (2)	0.0307 (7)	
N7	0.7762 (3)	0.2861 (2)	0.3071 (2)	0.0326 (6)	
H1n7	0.679039	0.198237	0.299084	0.0391*	
C4	0.9732 (3)	0.2570 (3)	0.3091 (2)	0.0320 (7)	
O2	1.1157 (2)	0.3698 (2)	0.3206 (2)	0.0481 (7)	
N8	0.9920 (3)	0.0979 (3)	0.2968 (2)	0.0448 (8)	
H1n8	1.1144 (13)	0.082 (3)	0.289 (3)	0.0537*	
H2n8	0.886 (2)	0.024 (2)	0.280 (3)	0.0537*	
N9	0.1970 (3)	0.1691 (2)	0.6254 (2)	0.0384 (7)	
H1n9	0.0709 (8)	0.174 (3)	0.621 (2)	0.0461*	
H2n9	0.237 (4)	0.0751 (15)	0.622 (2)	0.0461*	
N10	0.5220 (3)	0.2985 (3)	0.6358 (2)	0.0468 (8)	
H1n10	0.556 (3)	0.2016 (9)	0.626 (3)	0.0562*	
H2n10	0.613 (2)	0.3858 (13)	0.647 (3)	0.0562*	
C5	0.3284 (3)	0.3046 (3)	0.6375 (2)	0.0302 (7)	
N11	0.2757 (3)	0.4584 (2)	0.65242 (19)	0.0323 (6)	
H1n11	0.37363	0.545774	0.661465	0.0387*	
C6	0.0770 (3)	0.4880 (3)	0.6545 (2)	0.0316 (7)	
O3	−0.0658 (2)	0.3745 (2)	0.64508 (19)	0.0424 (6)	
N12	0.0598 (3)	0.6470 (2)	0.6695 (2)	0.0430 (7)	
H1n12	0.161 (2)	0.721 (2)	0.661 (3)	0.0516*	
H2n12	−0.0630 (13)	0.659 (3)	0.658 (3)	0.0516*	
Ow	0.8062 (3)	0.7323 (3)	−0.0104 (2)	0.0697 (8)	
H1ow	0.739 (4)	0.754 (5)	0.0580 (17)	0.1045*	
H2ow	0.741 (4)	0.740 (4)	−0.0799 (16)	0.1045*	

### Atomic displacement parameters (Å^2^)

**Table:**

	*U*^11^	*U*^22^	*U*^33^	*U*^12^	*U*^13^	*U*^23^
P1	0.0308 (3)	0.0250 (3)	0.0478 (3)	0.0036 (2)	−0.0033 (2)	0.0052 (2)
O11	0.0617 (12)	0.0372 (9)	0.0542 (11)	−0.0079 (8)	0.0044 (9)	0.0067 (8)
O12	0.0371 (9)	0.0255 (9)	0.0847 (12)	0.0056 (7)	0.0049 (8)	0.0106 (8)
O13	0.0397 (9)	0.0411 (9)	0.0739 (11)	0.0159 (7)	0.0033 (8)	0.0089 (8)
F1	0.0639 (10)	0.0506 (8)	0.0611 (8)	−0.0042 (7)	−0.0232 (7)	0.0090 (6)
P2	0.0257 (3)	0.0251 (3)	0.0541 (3)	0.0023 (2)	0.0003 (2)	0.0099 (2)
O21	0.0394 (9)	0.0446 (9)	0.0533 (9)	0.0170 (7)	−0.0010 (7)	0.0099 (7)
O22	0.0353 (10)	0.0307 (9)	0.1131 (16)	0.0079 (8)	−0.0088 (10)	0.0172 (9)
O23	0.0470 (11)	0.0692 (12)	0.0616 (11)	0.0002 (9)	−0.0090 (8)	−0.0062 (9)
F2	0.0487 (9)	0.0666 (10)	0.0979 (12)	−0.0042 (7)	0.0227 (8)	0.0303 (9)
N1	0.0317 (11)	0.0597 (14)	0.0576 (12)	0.0080 (10)	0.0003 (9)	0.0066 (11)
N2	0.0377 (11)	0.0354 (11)	0.0619 (12)	0.0039 (10)	−0.0026 (9)	0.0021 (9)
C1	0.0299 (11)	0.0441 (12)	0.0318 (10)	0.0044 (9)	0.0008 (8)	0.0041 (9)
N3	0.0301 (10)	0.0349 (10)	0.0460 (10)	−0.0020 (8)	−0.0006 (8)	0.0040 (8)
C2	0.0378 (13)	0.0445 (13)	0.0396 (11)	0.0082 (10)	0.0021 (9)	0.0047 (9)
O1	0.0280 (8)	0.0502 (10)	0.0799 (12)	0.0023 (8)	−0.0022 (8)	−0.0013 (8)
N4	0.0553 (15)	0.0443 (13)	0.0700 (14)	0.0132 (11)	0.0094 (12)	0.0138 (11)
N5	0.0301 (10)	0.0288 (11)	0.0668 (13)	0.0071 (9)	0.0014 (9)	0.0122 (9)
N6	0.0251 (11)	0.0400 (13)	0.0697 (13)	0.0082 (9)	0.0038 (9)	0.0130 (11)
C3	0.0240 (11)	0.0352 (12)	0.0337 (11)	0.0060 (9)	0.0026 (8)	0.0075 (9)
N7	0.0218 (9)	0.0258 (9)	0.0499 (10)	0.0022 (8)	0.0006 (8)	0.0082 (8)
C4	0.0233 (11)	0.0302 (13)	0.0435 (12)	0.0051 (10)	0.0006 (9)	0.0093 (10)
O2	0.0229 (8)	0.0362 (10)	0.0857 (13)	0.0036 (8)	0.0041 (8)	0.0140 (9)
N8	0.0314 (12)	0.0344 (12)	0.0686 (13)	0.0107 (10)	−0.0015 (10)	0.0057 (11)
N9	0.0277 (10)	0.0269 (11)	0.0605 (11)	0.0046 (9)	0.0011 (9)	0.0081 (9)
N10	0.0245 (10)	0.0463 (12)	0.0724 (14)	0.0098 (9)	0.0035 (10)	0.0146 (12)
C5	0.0265 (11)	0.0324 (12)	0.0315 (10)	0.0053 (9)	−0.0002 (8)	0.0056 (8)
N11	0.0221 (9)	0.0258 (10)	0.0475 (10)	0.0005 (8)	0.0011 (8)	0.0065 (8)
C6	0.0245 (11)	0.0324 (12)	0.0371 (11)	0.0040 (10)	0.0030 (9)	0.0051 (10)
O3	0.0216 (8)	0.0345 (9)	0.0698 (11)	0.0022 (7)	0.0026 (7)	0.0085 (8)
N12	0.0330 (12)	0.0287 (11)	0.0683 (13)	0.0067 (9)	0.0047 (10)	0.0106 (10)
Ow	0.0785 (14)	0.0877 (14)	0.0564 (11)	0.0446 (12)	0.0049 (10)	0.0221 (11)

### Geometric parameters (Å, °)

**Table:**

P1—O11	1.548 (2)	C6—O3	1.225 (3)
P1—O12	1.4752 (17)	C6—N12	1.323 (3)
P1—O13	1.4850 (17)	N1—H1n1	0.860 (18)
P1—F1	1.5603 (14)	N1—H2n1	0.860 (15)
O11—H1o11	0.80 (3)	N2—H1n2	0.860 (13)
P2—O21	1.5118 (18)	N2—H2n2	0.860 (16)
P2—O22	1.479 (2)	N3—H1n3	0.89
P2—O23	1.4949 (18)	N4—H1n4	0.860 (17)
P2—F2	1.5735 (18)	N4—H2n4	0.860 (19)
N1—C1	1.315 (3)	N5—H1n5	0.860 (8)
N2—C1	1.311 (3)	N5—H2n5	0.860 (19)
C1—N3	1.355 (3)	N6—H1n6	0.860 (17)
N3—C2	1.396 (3)	N6—H2n6	0.860 (15)
C2—O1	1.224 (3)	N7—H1n7	0.89
C2—N4	1.335 (3)	N8—H1n8	0.860 (12)
N5—C3	1.305 (3)	N8—H2n8	0.860 (15)
N6—C3	1.309 (3)	N9—H1n9	0.860 (8)
C3—N7	1.359 (3)	N9—H2n9	0.860 (16)
N7—C4	1.390 (3)	N10—H1n10	0.860 (11)
C4—O2	1.219 (3)	N10—H2n10	0.860 (11)
C4—N8	1.328 (3)	N11—H1n11	0.89
N9—C5	1.302 (3)	N12—H1n12	0.860 (17)
N10—C5	1.316 (3)	N12—H2n12	0.860 (13)
C5—N11	1.361 (3)	Ow—H1ow	0.82 (2)
N11—C6	1.401 (3)	Ow—H2ow	0.82 (2)
			
O11—P1—O12	113.89 (10)	C1—N3—H1n3	117.7615
O11—P1—O13	111.21 (11)	H1n3—N3—C2	117.7617
O11—P1—F1	98.53 (9)	C3—N7—H1n7	117.353
O12—P1—O13	116.89 (9)	H1n7—N7—C4	117.3532
O12—P1—F1	108.33 (9)	C5—N11—H1n11	117.8945
O13—P1—F1	105.98 (9)	C5—N11—C6	124.21 (17)
P1—O11—H1o11	110 (2)	H1n11—N11—C6	117.8946
O21—P2—O22	113.55 (10)	H1n1—N1—C1	122.1 (16)
O21—P2—O23	113.39 (11)	H2n1—N1—C1	118.5 (13)
O21—P2—F2	104.28 (10)	H1n2—N2—C1	115.7 (16)
O22—P2—O23	114.52 (13)	H2n2—N2—C1	121.3 (17)
O22—P2—F2	105.09 (11)	C2—N4—H1n4	118.9 (17)
O23—P2—F2	104.61 (10)	C2—N4—H2n4	118.4 (14)
N1—C1—N2	120.9 (2)	H1n5—N5—C3	120.6 (18)
N1—C1—N3	117.5 (2)	H2n5—N5—C3	116.5 (14)
N2—C1—N3	121.6 (2)	C4—N8—H1n8	112.8 (18)
C1—N3—C2	124.48 (18)	C4—N8—H2n8	118.8 (13)
N3—C2—O1	122.1 (2)	H1n9—N9—C5	119.9 (17)
N3—C2—N4	113.8 (2)	H2n9—N9—C5	119.7 (15)
O1—C2—N4	124.2 (2)	H1n10—N10—C5	116.9 (11)
N5—C3—N6	120.9 (2)	H2n10—N10—C5	123.1 (9)
N5—C3—N7	122.1 (2)	C6—N12—H1n12	121.4 (12)
N6—C3—N7	117.06 (19)	C6—N12—H2n12	111.6 (18)
C3—N7—C4	125.29 (17)	H1n1—N1—H2n1	119 (2)
N7—C4—O2	121.8 (2)	H1n2—N2—H2n2	123 (2)
N7—C4—N8	114.67 (18)	H1n4—N4—H2n4	120 (2)
O2—C4—N8	123.5 (2)	H1n5—N5—H2n5	123 (2)
N9—C5—N10	120.8 (2)	H1n6—N6—H2n6	117.9 (19)
N9—C5—N11	122.7 (2)	H1n8—N8—H2n8	127 (2)
N10—C5—N11	116.5 (2)	H1n9—N9—H2n9	120 (2)
C5—N11—C6	124.21 (17)	H1n10—N10—H2n10	120.0 (14)
N11—C6—O3	121.7 (2)	H1n12—N12—H2n12	123 (2)
N11—C6—N12	114.23 (18)	H1ow—Ow—H2ow	108 (3)
O3—C6—N12	124.0 (2)		
			
?—?—?—?	?		

### Hydrogen-bond geometry (Å, °)

**Table:**

*D*—H···*A*	*D*—H	H···*A*	*D*···*A*	*D*—H···*A*
O11—H1o11···O21	0.80 (3)	1.72 (4)	2.515 (3)	174 (4)
Ow—H1ow···O13	0.82 (2)	1.957 (19)	2.754 (3)	164 (2)
Ow—H2ow···O22^i^	0.82 (2)	2.253 (17)	3.037 (3)	160 (3)
Ow—H2ow···O23^i^	0.82 (2)	2.41 (3)	3.023 (3)	132 (3)
N1—H1n1···Ow	0.860 (18)	2.302 (15)	3.022 (4)	141 (2)
N1—H2n1···O1^ii^	0.861 (17)	2.24 (2)	2.795 (3)	122.4 (15)
N1—H2n1···O23^iii^	0.861 (15)	2.431 (14)	3.141 (3)	140.3 (18)
N6—H2n6···O2^ii^	0.860 (17)	2.23 (2)	2.750 (3)	119.0 (15)
N6—H2n6···O12^iv^	0.860 (17)	2.491 (15)	3.200 (3)	140.3 (17)
N6—H1n6···O11	0.861 (19)	2.28 (2)	3.141 (3)	174 (2)
N9—H2n9···O21^iv^	0.860 (17)	2.08 (2)	2.916 (2)	164 (2)
N9—H1n9···F2^v^	0.860 (7)	2.479 (19)	3.096 (2)	129 (2)
N9—H1n9···O3	0.860 (7)	2.01 (2)	2.634 (2)	129 (2)
N2—H2n2···Ow	0.861 (16)	2.12 (2)	2.894 (3)	150 (2)
N2—H1n2···O1	0.861 (12)	1.94 (2)	2.618 (3)	135 (2)
N5—H2n5···O13	0.862 (19)	2.07 (2)	2.906 (3)	165 (2)
N5—H1n5···O2	0.860 (7)	2.03 (2)	2.646 (3)	128 (2)
N10—H1n10···O21^iv^	0.860 (13)	2.595 (10)	3.318 (3)	142.5 (17)
N10—H2n10···O3^vi^	0.861 (13)	2.186 (14)	2.750 (3)	122.9 (11)
N10—H2n10···O22	0.861 (13)	2.524 (13)	3.209 (3)	137.1 (11)
N3—H1n3···O23^iii^	0.89	1.95	2.770 (3)	153
N7—H1n7···O12^iv^	0.89	1.93	2.807 (3)	166
N11—H1n11···O22	0.89	1.93	2.804 (3)	166
N4—H1n4···O23^vii^	0.860 (14)	2.386 (16)	3.170 (3)	152 (2)
N4—H2n4···Ow^iv^	0.86 (2)	2.32 (2)	3.173 (3)	176 (2)
N8—H2n8···O13^iv^	0.857 (16)	2.024 (16)	2.877 (3)	173.8 (15)
N8—H1n8···O12^viii^	0.860 (10)	2.179 (10)	3.034 (3)	173 (3)
N12—H1n12···O21	0.859 (11)	2.115 (16)	2.972 (2)	175 (3)
N12—H2n12···O22^ii^	0.859 (11)	2.198 (11)	3.031 (3)	163 (3)

Symmetry codes:  (i) *x*, *y*, *z*−1; (ii) *x*−1, *y*, *z*; (iii) *x*, *y*−1, *z*−1; (iv) *x*, *y*−1, *z*; (v) *x*−1, *y*−1, *z*; (vi) *x*+1, *y*, *z*; (vii) *x*+1, *y*−1, *z*−1; (viii) *x*+1, *y*−1, *z*.

### Table 3 Table 2. The hydrogen-bond pattern in the title structure. The atoms N1-N4, N5-N8 and N9-N12 are situated in the first (Fig. 3), second (Fig. 4) and the third layer (Fig. 5), respectively. The atoms in the blocks comprise the corresponding atoms in the respective layers: *e.g.* N2—H2n2···O1, N5—H2n5···O2, N10—H1n10···O3.

**Table:**

D-H···A	D-H (Å)	H···A (Å)	D···A (Å)	D-H···A (°)	sym.
O11-H1o11···O21	0.80 (4)	1.72 (4)	2.515 (3)	174 (4)	
Ow-H1ow···O13	0.82 (2)	1.957 (19)	2.754 (3)	164 (2)	
Ow-H2ow···O22	0.82 (2)	2.253 (17)	3.037 (3)	160 (3)	x, y, -1+z
Ow-H2ow···O23	0.82 (2)	2.41 (3)	3.023 (3)	132 (3)	x, y, -1+z
					
N1-H1n1···Ow	0.860 (18)	2.302 (15)	3.022 (4)	141 (2)	
N1-H2n1···O1	0.861 (17)	2.24 (2)	2.795 (3)	122.4 (15)	-1+x, y, z
N1-H2n1···O23	0.861 (15)	2.431 (14)	3.141 (3)	140.3 (18)	x, -1+y, -1+z
N6-H2n6···O2	0.860 (17)	2.23 (2)	2.750 (3)	119.0 (15)	-1+x, y, z
N6-H2n6···O12	0.860 (17)	2.491 (15)	3.200 (3)	140.3 (17)	
N6-H1n6···O11	0.861 (19)	2.28 (2)	3.141 (3)	174 (2)	
N9-H2n9···O21	0.860 (17)	2.08 (2)	2.916 (2)	164 (2)	x, -1+y, z
N9-H1n9···F2	0.860 (7)	2.479 (19)	3.096 (2)	129 (2)	-1+x, -1+y, z
N9-H1n9···O3	0.860 (7)	2.01 (2)	2.634 (2)	129 (2)	
					
N2-H2n2···Ow	0.861 (16)	2.12 (2)	2.894 (3)	150 (2)	
N2-H1n2···O1	0.861 (12)	1.94 (2)	2.618 (3)	135 (2)	
N5-H2n5···O13	0.862 (19)	2.07 (2)	2.906 (3)	165 (2)	
N5-H2n5···O2	0.860 (7)	2.03 (2)	2.646 (3)	128 (2)	
N10-H1n10···O21	0.860 (13)	2.595 (10)	3.318 (3)	142.5 (17)	x, -1+y, z
N10-H2n10···O3	0.861 (13)	2.186 (14)	2.750 (3)	122.9 (11)	1+x, y, z
N10-H2n10···O22	0.861 (13)	2.524 (13)	3.209 (3)	137.1 (11)	
					
N3-H1n3..O23	0.89	1.95	2.770 (3)	153	x,-1+y,-1+z
N7-H1n7···O12	0.89	1.93	2.807 (3)	166	x,-1+y,z
N11-H1n11 O22	0.89	1.93	2.804 (3)	166	
					
N4-H1n4···O23	0.860 (14)	2.386 (16)	3.170 (3)	152 (2)	1+x, -1+y, -1+z
N4-H2n4···Ow	0.86 (2)	2.32 (2)	3.173 (3)	176 (2)	x, -1+y, z
N8-H1n8···O13	0.857 (16)	2.024 (16)	2.877 (3)	173.8 (15)	x, -1+y, z
N8-H2n8···O12	0.860 (10)	2.179 (10)	3.034 (3)	173 (3)	1+x, -1+y, z
N12-H1n12···O21	0.859 (11)	2.115 (16)	2.972 (2)	175 (3)	
N12-H2n12···O22	0.859 (11)	2.198 (11)	3.031 (3)	163 (3)	-1+x, y, z
